# Reconstruction of xylose utilization pathway and regulons in Firmicutes

**DOI:** 10.1186/1471-2164-11-255

**Published:** 2010-04-21

**Authors:** Yang Gu, Yi Ding, Cong Ren, Zhe Sun, Dmitry A Rodionov, Weiwen Zhang, Sheng Yang, Chen Yang, Weihong Jiang

**Affiliations:** 1 Key Laboratory of Synthetic Biology, Institute of Plant Physiology and Ecology, Shanghai Institutes for Biological Sciences, Chinese Academy of Sciences, Shanghai 200032, China; 2 Burnham Institute for Medical Research, La Jolla, California 92037; 3 Institute for Information Transmission Problems, Russian Academy of Sciences, Moscow 127994, Russia; 4 Center of Ecogenomics, Biodesign Institute, Arizona State University, Tempe, Arizona 85287-6501, USA; 5 Shanghai Research and Development Center of Industrial Biotechnology, Shanghai 201201, China

## Abstract

**Background:**

Many Firmicutes bacteria, including solvent-producing clostridia such as *Clostridium acetobutylicum*, are able to utilize xylose, an abundant carbon source in nature. Nevertheless, homology searches failed to recognize all the genes for the complete xylose and xyloside utilization pathway in most of them. Moreover, the regulatory mechanisms of xylose catabolism in many Firmicutes except *Bacillus *spp. still remained unclear.

**Results:**

A comparative genomic approach was used to reconstruct the xylose and xyloside utilization pathway and analyze its regulatory mechanisms in 24 genomes of the Firmicutes. A novel xylose isomerase that is not homologous to previously characterized xylose isomerase, was identified in *C. acetobutylicum *and several other Clostridia species. The candidate genes for the xylulokinase, xylose transporters, and the transcriptional regulator of xylose metabolism (XylR), were unambiguously assigned in all of the analyzed species based on the analysis of conserved chromosomal gene clustering and regulons. The predicted functions of these genes in *C. acetobutylicum *were experimentally confirmed through a combination of genetic and biochemical techniques. XylR regulons were reconstructed by identification and comparative analysis of XylR-binding sites upstream of xylose and xyloside utilization genes. A novel XylR-binding DNA motif, which is exceptionally distinct from the DNA motif known for *Bacillus *XylR, was identified in three Clostridiales species and experimentally validated in *C. acetobutylicum *by an electrophoretic mobility shift assay.

**Conclusions:**

This study provided comprehensive insights to the xylose catabolism and its regulation in diverse Firmicutes bacteria especially Clostridia species, and paved ways for improving xylose utilization capability in *C. acetobutylicum *by genetic engineering in the future.

## Background

The Firmicutes (Bacilli/Clostridia) are a diverse group of Gram-positive bacteria that includes a large number of species that produce lactic acid, acetone, butanol, and ethanol through fermentation of a variety of carbon sources. Many of these bacteria were originally isolated from the plant environments such as garden soil, fruits, and vegetables [1,2]. Among them, *Clostridium acetobutylicum *is one of the best-studied clostridia and was used to develop an industrial fermentation process for producing solvents [3,4]. This strain is known to utilize a broad range of monosaccharides, disaccharides, starches, and other substrates such as whey and xylan [5,6].

Xylan and xyloglucan, the major hemicellulose components of plant cell walls, are two of the most abundant polysaccharides in nature and play an important role in supplying carbon and energy to a variety of organisms [7,8]. Depolymerization of xylan and xyloglucan produces β- and α-xylosides, respectively, that are transported into the cell and further degraded into D-xylose [9]. D-Xylose is finally transformed to the common metabolic intermediate xylulose 5-phosphate. Genetics and regulation of the xylanolytic machinery have been studied in some model species of Bacilli class, such as *Bacillus subtilis *[10] and *Lactococcus lactis *[11], but so far not in Clostridia.

**Figure 1:**
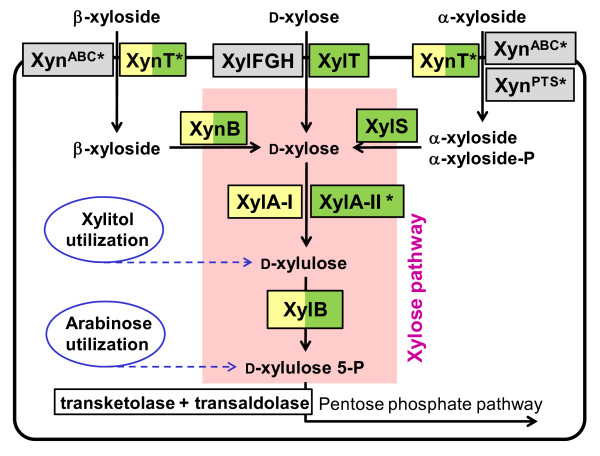
**Reconstruction of the xylose and xyloside utilization pathway in Firmicutes**. Functional roles present in *C. acetobutylicum *and *B. subtilis *are shown on green and yellow backgrounds, respectively. Those present in other bacteria of the same lineage (but not in *C. acetobutylicum *and *B. subtilis*) are shown on a gray background. Tentatively predicted functional roles are marked by asterisks. Solid arrows denote enzymatic reactions and transport, and broken arrows denote links to other catabolic pathways (utilization of arabinose and xylitol) that are not analyzed in this study.

**Figure 2:**
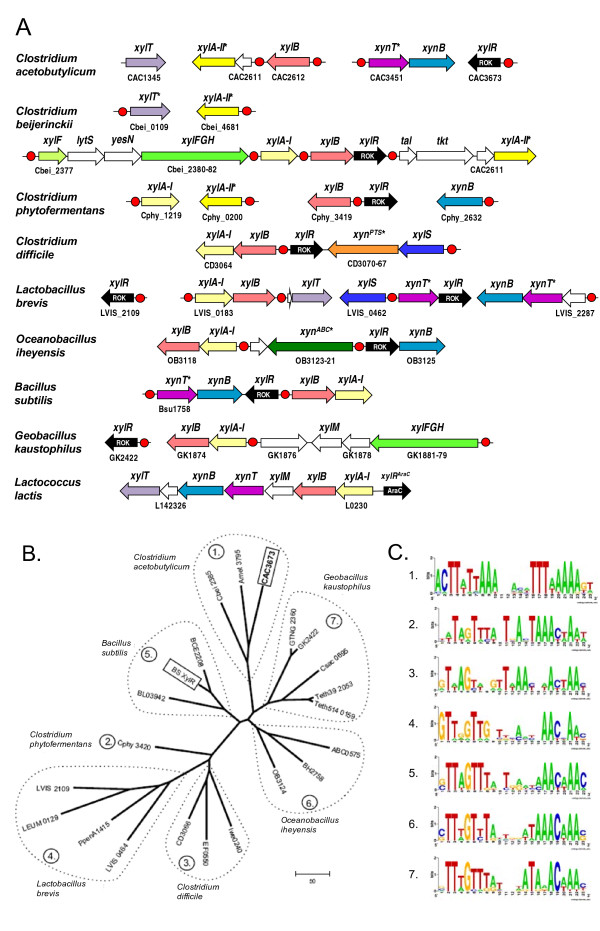
**Genomic context of genes associated with xylose and xyloside utilization in Firmicutes**. (A) Examples of chromosomal clusters and putative regulons containing genes involved in xylose and xyloside utilization. Candidate regulatory sites of XylR from ROK family are shown by red circles. Genes predicted by genome context analysis are marked by asterisks. Homologous genes are marked by matching colors. (B) Phylogenetic tree and (C) DNA recognition motifs of XylR including all known and predicted xylose regulators from Firmicutes. XylR proteins recognizing the same DNA motif are grouped, and the corresponding motif sequence logos are shown in (C).

In bacteria, the transformation of D-xylose to xylulose 5-P is catalyzed via consecutive isomerization to D-xylulose and phosphorylation reactions (Figure 1). This two-step biochemical pathway appears to be conserved in both non-xylanolytic bacteria (such as *Escherichia coli*) and xylanolytic bacteria such as *B. subtilis *[12,13]. In *B. subtilis*, the genes involved in the xylose and xyloside utilization pathway are clustered into two operons, *xylAB *and *xynTB *(Figure 2). Their expression is negatively regulated at the transcriptional level by the regulator XylR [14]. Due to the lack of a xylose uptake system, *B. subtilis *is unable to grow with xylose as a sole carbon source [15].

Several *Clostridium *species have been shown to metabolize D-xylose by early studies and our preliminary analysis [16,17]. However, the initial genomic survey of *C. acetobutylicum *ATCC 824 identified only the gene encoding xylulokinase in the xylose pathway, whereas the ortholog of the *xylA *gene encoding xylose isomerase was not found [4]. Although several genes of xyloside metabolism are annotated in public databases (*e.g*. GenBank or KEGG), some of these annotations are imprecise and have not been consistently projected across all the sequenced clostridia. Moreover, our current knowledge of transcriptional regulation of xylose utilization pathway in Gram-positive bacteria was limited to *Bacillus *spp. This prompted us to perform a detailed analysis of xylose utilization and its regulatory mechanisms in the species of Bacilli and Clostridia classes by combining comparative genomic analyses with genetic and biochemical techniques.

In this study, we used a subsystems-based comparative genomic analysis [18,19] to explore the xylose and xyloside utilization machinery in the Firmicutes species with completely sequenced genomes. A novel xylose isomerase (named XylA-II) that is not homologous to previously characterized XylA, was identified in several Clostridia species (*e.g*. CAC2610). In *C. acetobutylicum *the xylose utilization pathway also includes a xylulokinase (XylB, CAC2612), a xylose proton-symporter (XylT, CAC1345), and a transcriptional regulator (XylR, CAC3673). The predicted functions of these genes in *C. acetobutylicum *were experimentally confirmed through a combination of genetic and biochemical techniques. We conclusively showed that the identified gene *xylA-II *encodes a fully functional xylose isomerase that catalyze the transformation of D-xylose to D-xylulose. In addition, we have also tentatively identified several other genes likely associated with the utilization of β- or α-xyloside.

Many of these genes occurred in operons that formed a predicted regulon controlled by XylR. Comparative analysis of upstream regions of the xylose and xyloside utilization genes allowed identification of candidate DNA motifs for various groups of XylR regulators and reconstruction of XylR regulons. A novel XylR-binding DNA motif, which is exceptionally distinct from the DNA motif known for *Bacillus *XylR, was identified in three Clostridiales species and experimentally validated in *C. acetobutylicum *by an electrophoretic mobility shift assay (EMSA).

## Results

### (i) Comparative genomics of xylose and xyloside utilization in Firmicutes

**Figure 3:**
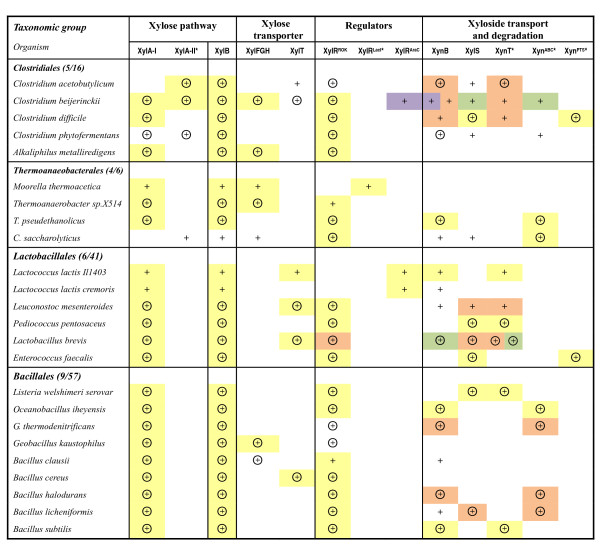
**Occurrence and features of genes involved in xylose and xyloside utilization pathway in Firmicutes**. Species with completely sequenced genomes in Bacilli/Clostridia classes are shown as rows. The presence of genes for the respective functional roles is shown by "+". Genes clustered on the chromosome are marked by the same background color. Candidate XylR regulon members are circled. Tentatively predicted functional roles are marked by asterisks. The number of species with xylose pathway divided by the total number of genomes in each taxonomic group is shown in parenthesis.

The subsystems-based approach was used to assess metabolic potential of Firmicutes species with completely sequenced genomes in utilization of xylose and its oligomeric precursors. For the twenty-four species that possess the xylose pathway genes, we reconstructed the xylose and xyloside utilization pathway and analyzed its regulatory mechanisms. The detailed results of this analysis are captured in the SEED subsystem available on line and in Additional file 1. The key results are illustrated in Figure 3 and Table 1 and contain both previously known and novel features predicted using the genome context analysis. Some of these features are briefly highlighted below where we focus mostly on novel findings and conjectures.

**Table 1:** Predicted functional assignments in the xylose and xyloside utilization pathway

Protein^a^	Example gene ID	Predicted functional role	Annotation in GenBank	Phylogenetic distribution
**XylA-II**	CAC2610	Xylose isomerase	Fucose isomerase related protein	*Clostridium*, *C. saccharolyticus*
**XylB**	CAC2612	Xylulokinase	Xylulokinase (with CAC1344)	Bacilli, Clostridia
**XylT**	Cbei_0109	Xylose H^+^-symporter	Sugar transporter	*Clostridium*, Lactobacillales
**XylR**	CAC3673	Xylose regulator	XylR regulator (with CAC0933)	Bacilli, Clostridia
XynT	CAC3451	Xyloside Na^+^(H^+^)**-**symporter	Sugar Na^+^(H^+^)-symporter	*Clostridium*, Lactobacillales, Bacillales
Xyn^ABC^	OB3123-3121	Xyloside ABC transporter	Sugar ABC transporter	*Clostridium*, Thermoanaeobacterales, Bacillaceae
Xyn^PTS^	CD3070-3067	Xyloside PTS transporter	PTS system	*C. difficile*, *E. faecalis*

^a ^Predicted functional assignments that were experimentally verified in *C. acetobutylicum *are marked by bold type and underlined.

#### XylR regulon

The transcriptional factor XylR belongs to ROK (Repressor, Open reading frame, Kinase) protein family and has an N-terminal DNA-binding domain and a C-terminal sugar kinase-like domain [20]. Based on homology supported by chromosomal clustering with xylose pathway genes, we identified XylR orthologs in most genomes of Firmicutes (Figure 3 and Additional file 1). Although XylR has been characterized in *Bacillus *spp. as a repressor of the *xyl *operon encoding xylose pathway genes [12], the annotations of XylR homologs in public databases are incomplete and imprecise due to the presence of functionally divergent paralogs and limited experimental data on their characterization. For example, *xylR *is assigned to four genes in *C. acetobutylicum *genome (*i.e*. CAC3673, CAC0933, CAC1086, and CAC0183) in the public databases (*e.g*. KEGG). Only CAC3673 was deemed to xylose regulator in this study, because it displayed a closest homology with the characterized XylR from *B. subtilis *(31% *vs*. <22% identity). Moreover, the close orthologs of CAC3673 in *Clostridium beijerinckii *and *Alkaliphilus metalliredigens *(Cbei_2385 and Amet_3795) are clustered on the chromosome with xylose pathway genes (Figure 3).

The phylogenetic tree was constructed for 22 representative XylR proteins from Firmicutes including two paralogs in *Lactobacillus brevis*, where at least 7 branches were observed on the phylogenetic tree of XylR proteins (Figure 2B). The lowest pairwise sequence similarity between different groups of XylR proteins was 21% (between XylR from *C. acetobutylicum *and *Leuconostoc mesenteroides*). XylR from *C. acetobutylicum *was in the same group with orthologous proteins from *C. beijerinckii*, and *A. metalliredigens*. This group of clostridial XylR proteins stands alone from XylR orthologs in other *Clostridium *such as *C. phytofermentans *and *C. difficile*.

Although the XylR regulons in Bacilli/Clostridia have been analyzed previously [21], a rapidly growing number of complete genomes in these classes allowed significant improvement of the accuracy of XylR-binding DNA motifs and expansion of XylR regulons. Analysis of upstream regions of XylR-controlled genes and their orthologs in XylR-encoding genomes resulted in identification of the group-specific XylR-binding DNA motifs that were used to search for additional candidate

XylR-binding sites in the analyzed groups of genomes (Figure 2C). The obtained consensus sequences for XylR-binding sites in the Bacillaceae genomes (groups 5, 6 and 7 on the XylR protein tree) were in accordance with that experimentally determined for *B. subtilis *XylR [22]. Predicted DNA motifs of XylR regulators from other groups on the tree were partially similar to that from the Bacillaceae. However, the group 1 of XylR regulators from *C. acetobutylicum*, *C. beijerinckii*, and *A. metalliredigens *has an exceptionally different DNA recognition motif, a 25-bp inverted repeat with consensus sequence 5'-ACTTattAAAnnnnnTTTaaAAAgt-3' (Figure 2C).

The identified candidate DNA-binding sites of different groups of XylR regulators were detected in the promoter regions of most xylose utilization genes in Firmicutes (Figure 3 and Additional file 2). The most conserved part of the XylR regulon includes the xylose pathway genes *xylA *and *xylB *and transporter genes *xylFGH *or *xylT*. The XylR regulon members also include the genes involved in uptake and degradation of α-, and β-xylosides. In addition, the presence of XylR-binding sites upstream of the *xylR *gene in many species suggests possible autoregulation of its expression. Hence, assignment of the gene *xylR *(*e.g*. CAC3673) is further supported by sharing upstream XylR-binding sites with xylose pathway genes. Remarkably, a XylR-binding site in the *C. acetobutylicum *and several other *Clostridium *genomes was detected upstream of a hypothetical gene encoding a novel non-orthologous variant of xylose isomerase characterized in this work (see below).

Orthologs of XylR regulator were not found in several species of Firmicutes possessing a complete version of the xylose utilization pathway, *i.e*., *Moorella thermoacetica *and *L. lactis*. In the first species, a hypothetical LacI-type transcriptional factor (Moth_2024) was inferred based on chromosomal clustering with the xylose pathway genes (Figure 3). The xylose operon in *L. lactis *is known to be controlled by an AraC-type regulator (also called XylR) [23]. Since there are only few genomes encoding these two types of xylose regulator, their DNA recognition motifs could not be determined accurately. Overall, alternative transcriptional regulators of xylose metabolism appear to be present in various species of Firmicutes.

#### Xyloside uptake and degradation

The analysis of operons and regulons associated with xylose and xyloside utilization subsystem allowed us to accurately annotate and map some previously uncharacterized components of xyloside utilization machinery in Firmicutes. Most genes encoding β- and α-xylosidases (*xynB *and *xylS*, respectively) appear to be clustered on the chromosome and/or co-regulated with the xylose pathway genes (Figure 3).

Based on the genome context analysis, we predicted the involvement of three types of transporters, XynT, Xyn^ABC^, and Xyn^PTS^, in the xyloside uptake (Figure 3). XynT belongs to the MFS (Major Facilitator Superfamily) transporter family. The *xynT *gene was identified as a member of the XylR regulon in *C. acetobutylicum *(CAC3451) and several Lactobacillales and Bacillales (designated as *xynP *in *B. subtilis *by [24]). It is also positionally clustered with xylosidase and xylose pathway genes (Figure 3). An alternative system of xyloside transport via a committed ABC cassette was predicted for *Oceanobacillus iheyensis *(OB3123-OB3121) and several other species. The Xyn^ABC ^transport system is homologous to an oligosaccharide ABC transporter from the *Streptococcus mutans *(~24% identity; [25]). The functional prediction is supported by chromosomal clustering and by sharing upstream XylR-binding sites with xylosidase and other *xyl *genes (Figure 2A). A novel xyloside transporter from the phosphotransferase (PTS) system family was predicted for *Enterococcus faecalis *and *C. difficile*. The gene cluster encoding this Xyn^PTS ^system (e.g. CD3070-CD3067) is a candidate member of the predicted XylR regulons and co-localized with α-xylosidase gene (*xylS*) in both species (Figure 2A), suggesting involvement of Xyn^PTS ^in uptake of α-xyloside.

#### Xylose transport

The ABC-type xylose transporter XylFGH was originally described in *E. coli *[26]. An orthologous xylose ABC transporter was found in the genomic context of the xylose utilization genes in several Clostridiales, Thermoanaerobacterales, and Bacillales species (*e.g*. Cbei_2380-Cbei_2382 in *C. beijerinckii*; see Figure 3). Another xylose transporter belonging to the MFS transporter family, XylT, was described in *Bacillus megaterium *[27] and *L. brevis *[28]. Its orthologs in several species of Bacilli/Clostridia have been annotated as xylose proton-symporter in the public databases (*e.g*. CAC1345 of *C. acetobutylicum*). In this study XylT was tentatively identified in more species such as *C. beijerinckii*. This functional assignment is supported by the conserved co-localization on the chromosome and by predicted co-regulation (via upstream XylR-binding sites) with other *xyl *genes (Figure 3).

#### Xylose pathway

Xylose isomerase (EC 5.3.1.5) is required for the first reaction of xylose utilization, converting D-xylose into D-xylulose. This enzyme, a product of the *xylA *gene, was characterized in detail in *L. lactis *[29], and its orthologs are present in many bacteria including *B. subtilis*. However, analysis of the xylose utilization subsystem showed that this gene is missing in *C. acetobutylicum *and *Caldicellulosiruptor saccharolyticus *that have other components of the xylose utilization pathway. Based on genomic and functional context analysis (Figure 3), we have tentatively identified a candidate gene for an alternative xylose isomerase (termed here XylA-II), which is not homologous to XylA from *B. subtilis *and other Firmicutes (called here as XylA-I). XylA-II belongs to the fucose isomerase FucI family (PF02952), with a weak ~14% similarity to FucI from *E. coli *[30]. The *xylA-II *gene in *C. acetobutylicum *(CAC2610) and *C. beijerinckii *(Cbei_2389) is clustered with *xylB *and preceded by candidate XylR-binding sites (Figure 2A), whereas *C. phytofermentans *has a separate *xylA-II *gene preceded by a candidate XylR site.

Xylulokinase (EC 2.7.1.17), XylB, is required for the phosphorylation of D-xylulose yielding D-xylulose 5-P, a key intermediate in the pentose phosphate pathway of the central carbon metabolism. XylB, originally characterized in *E. coli *[26], is the invariant component of the xylose pathway (Figure 3). However, accurate functional assignment of sugar kinase genes such as *xylB *is not easy due to the presence of functionally divergent paralogs. For example, both CAC2612 and CAC1344 in *C. acetobutylicum *genome are annotated as xylulokinase in GenBank based on homology. Here only CAC2612 and its orthologs were identified as XylB based on the genome context evidence as follows: chromosomal clustering with xylose utilization genes and assignment to the XylR regulon (Figure 3).

In summary, the subsystem reconstruction and the genome context analysis in 24 genomes of Firmicutes allowed us to predict the candidate genes for a novel xylose isomerase and a xylulokinase as well as for previously uncharacterized xylose and xyloside transporters and xylose regulator (Table 1). The regulon analysis led to tentative identification of different types of DNA motifs that correspond to the binding sites of transcriptional regulator XylR. In the second part of this study, we have performed experimental validation of the predicted functions of *xylA-II*, *xylB*, *xylT*, and *xylR *genes as well as the identified XylR-binding sites in *C. acetobutylicum*.

### (ii) Experimental validation

#### Mutagenesis corroborates predicted xylose pathway genes

**Figure 4:**
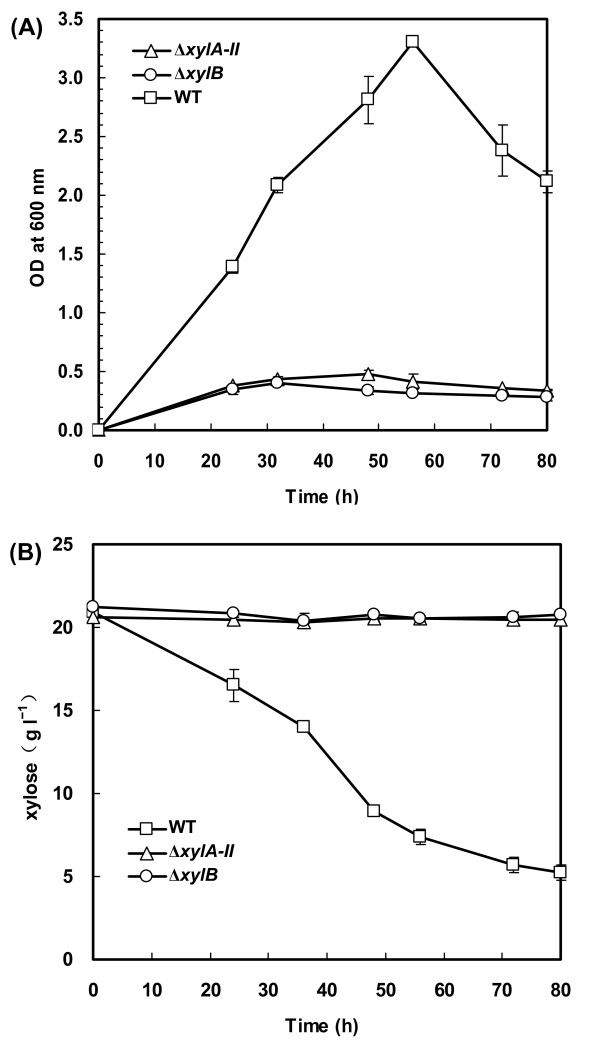
**Effect of *xylA-II *or *xylB *disruption on cell growth and xylose utilization of *C. acetobutylicum***. Cells were grown in the medium containing 20 g l^-1 ^of D-xylose as the sole carbon source. The optical density at 600 nm (A) and xylose concentration in the medium (B) were monitored. Data shown are means ± standard deviations calculated from triplicate individual experiments.

To validate the role of the inferred xylose pathway genes in *C. acetobutylicum*, we disrupted the respective genes by inserting an intron (confirmed by PCR as shown in Additional file 3) and tested the ability of these mutants to grow on xylose as the sole carbon source. Inactivation of the gene encoding putative xylose isomerase (Δ*xylA-II*) or xylulokinase (Δ*xylB*) abolished the growth of the resulting strains on xylose (Figure 4A) whereas their growth on glucose was not impaired (data not shown). The mutants did not consume any xylose after a 80-h incubation in the minimal medium containing 20 g l^-1 ^of xylose (Figure 4B). Therefore, the phenotypes of *C. acetobutylicum *mutants were consistent with the predictions of the bioinformatics analysis and confirmed the predicted physiological roles of *xylA-II *and *xylB *in utilization of xylose.

#### Heterologous-host complementation supports functional assignments of predicted xylose pathway genes

**Figure 5:**
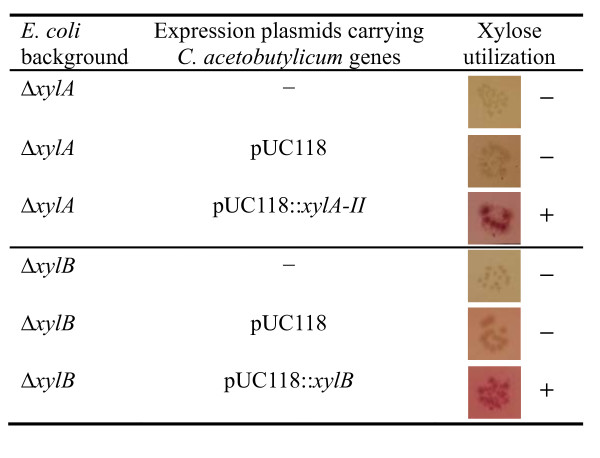
**Complementation of *E. coli *mutants deficient in xylose isomerase or xylulokinase by *C. acetobutylicum *genes**. The empty vector was expressed in the same strain as a negative control. The ability of xylose utilization was tested using MacConkey agar supplemented with 10 g l^-1 ^xylose. The colonies of cells with the ability in xylose utilization showed red color, whereas the colonies of cells unable to utilize xylose keep yellow.

To test whether the identified genes are not only required but also sufficient to perform the predicted functions, we carried out genetic complementation experiments. Plasmid constructs containing *C. acetobutylicum *genes *xylA-II *and *xylB *were introduced into *E. coli *K-12 mutants deficient in xylose isomerase (Δ*xylA*) or xylulokinase (Δ*xylB*). The resulting strains were tested for the ability of xylose utilization using MacConkey agar supplemented with xylose as a carbon source (Figure 5). Expression of the *C. acetobutylicum xylA-II *gene completely restored the ability of *E. coli *Δ*xylA *mutant to utilize xylose. Similarly, expression of *xylB *from *C. acetobutylicum *successfully complemented the xylulokinase deficiency and restored the ability of *E. coli *Δ*xylB *mutant in xylose utilization (Figure 5). On the other hand, expression of the gene CAC1344, which is also annotated as xylulokinase in GenBank, had no appreciable effect on the impaired xylose utilization of Δ*xylB *mutant (data not shown). Our results indicate that *xylA-II *and *xylB *of *C. acetobutylicum *encode active enzymes functionally equivalent to xylose isomerase and xylulokinase, respectively.

#### *In vitro *activity of the novel xylose isomerase and xylulokinase

To extend the genetic findings and provide biochemical evidence to the proposed gene assignments, we used the recombinant XylA-II and XylB from *C. acetobutylicum*, which were overexpressed in *E. coli *with the N-terminal His_6 _tag and purified using Ni-NTA affinity chromatography, to test for xylose isomerase and xylulokinase activities, respectively. Expected enzymatic activities of both proteins were verified using the specific assays described in Materials and Methods. The specific activity of the *C. acetobutylicum *XylA-II was 1.90 ± 0.14 μmol mg^-1 ^min^-1^, which is comparable with the respective values reported for the enzyme from *E. coli *(0.87 μmol mg^-1 ^min^-1^) [31] while lower than that for *Bacillus licheniformis *XylA (22.2 μmol mg^-1 ^min^-1^) [32]. The *C. acetobutylicum *XylB displayed a xylulokinase activity, although the specific activity value (3.04 ± 0.13 μmol mg^-1 ^min^-1^) is significantly lower than that of *E. coli *XylB (298 μmol mg^-1 ^min^-1^) [33]. Therefore, the biochemical activity assays provided an independent verification of the predicted enzymatic activities of XylA-II and XylB from *C. acetobutylicum*.

#### Experimental assessment of the predicted xylose transporter gene

**Figure 6:**
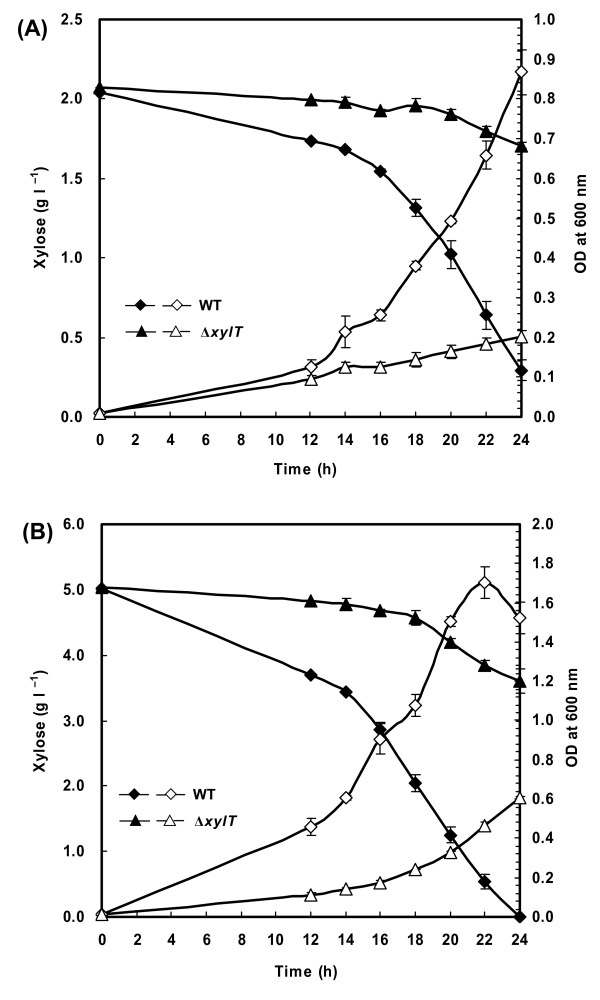
**Effect of *xylT *disruption on cell growth and xylose utilization of *C. acetobutylicum***. Cells were grown in the medium containing 2 g l^-1 ^(A) or 5 g l^-1 ^(B) of D-xylose as the sole carbon source. Open symbols, optical density at 600 nm; filled symbols, xylose concentration in the medium. Data shown are means ± standard deviations calculated from triplicate individual experiments.

To test the role of the inferred xylose transporter gene in *C. acetobutylicum*, we disrupted the gene CAC1345 (*xylT*) by inserting an intron (confirmed by PCR as shown in Additional file 3) and assessed the effect of its inactivation on cell growth and xylose consumption. A significant impairment of cell growth on xylose was observed for Δ*xylT *mutant compared to the wild-type strain (Figure 6) whereas their growth on glucose was not affected (data not shown). In the medium containing 2 g l^-1 ^of xylose, specific growth rate of Δ*xylT *mutant (0.06 h^-1^) was more than three-fold lower than that of wild-type strain (0.20 h^-1^). After a 20-h incubation on 2 g l^-1 ^of xylose, about 50% of xylose was consumed by the wild-type strain, whereas the Δ*xylT *mutant consumed less than 8% of xylose (Figure 6A). The difference between the growth curves was smaller at high concentrations of xylose (e.g. 50 g l^-1^; data not shown). These observations suggest that *xylT *contributes to the uptake of xylose in *C. acetobutylicum*. The obtained results also revealed that in addition to XylT, *C. acetobutylicum *must have another, hitherto-unknown transport system for xylose uptake.

**Figure 7:**
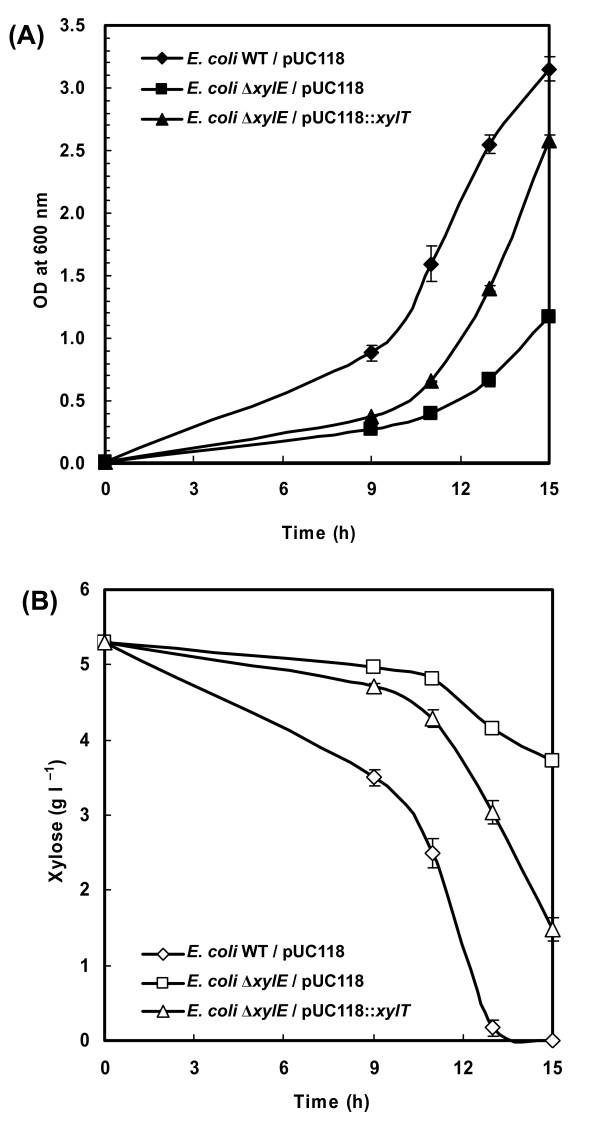
**An accelerated xylose utilization of *E. coli *Δ*xylE *mutant carrying *C. acetobutylicum xylT *gene**. Cells were grown in the minimal medium containing 5 g l^-1 ^of D-xylose as the sole carbon source. The optical density at 600 nm (A) and xylose concentration in the medium (B) were monitored. *E. coli *Δ*xylE *mutant strain with an empty vector was used a control. Data shown are means ± standard deviations calculated from triplicate individual experiments.

An additional verification of the xylose transporter gene was obtained using the genetic complementation experiment. A plasmid construct containing *C. acetobutylicum xylT *gene was introduced into *E. coli *K-12 Δ*xylE *mutant that lacks xylose proton-symporter while still has the ABC transporter system for xylose (XylFGH). The specific growth rate of this engineered strain (0.36 h^-1^) was higher than that of the control strain carrying an empty vector plasmid (0.25 h^-1^) when both strains were grown in the minimal medium containing 5 g l^-1 ^of xylose (Figure 7A). Concomitantly, expression of *C. acetobutylicum xylT *led to an acceleration in xylose consumption (Figure 7B), indicating that the *xylT *gene product is able to transport xylose. Overall, the cumulative evidence generated in the two different types of experiments described above provided strong support for the tentative gene assignment of the xylose transporter in *C. acetobutylicum*.

#### Experimental testing of xylose regulator binding to predicted DNA targets

**Figure 8:**
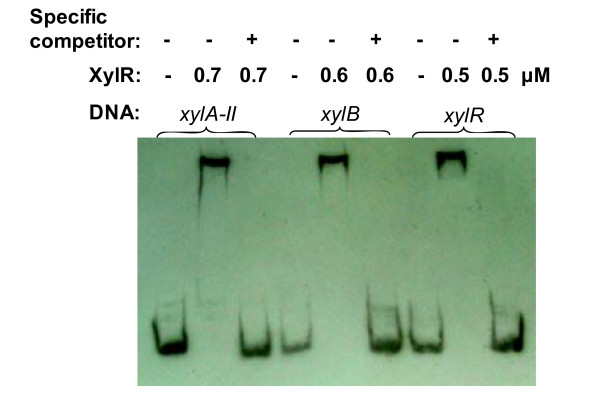
**EMSA to assess the interactions of *C. acetobutylicum *XylR with their cognate DNA signals**. Each of the three 180-bp target DNA fragments (1 nM) from the upstream region of CAC2611-*xylA-II*, *xylB*, and *xylR *genes, respectively, was incubated for 20 min at 28°C with or without 0.5-0.7 μM of the XylR protein. Specificity of the XylR-DNA interactions was tested by competition with 0.4 μM of non-biotinylated target DNA (specific competitor). Salmon sperm DNA (2 μg) was added to all binding reaction mixtures as a non-specific competitor.

The ability of XylR protein to specifically bind to the predicted DNA sites was tested by EMSA using the purified recombinant XylR protein from *C. acetobutylicum*. The predicted XylR-binding sites in *C. acetobutylicum *and several other species represent a distinct DNA motif from that known for *Bacillus *XylR (as described in the previous section). Three predicted target DNA fragments from the upstream regions of *C. acetobutylicum xylA-II*, *xylB*, and *xylR *genes, respectively, were used in EMSA. A substantial shift of the DNA band was observed in all three cases upon incubation of XylR protein with the target DNA fragments (Figure 8). A typical protein concentration dependence of DNA-binding is illustrated in Additional file 4 showing increasing intensity of the shifted DNA band in the presence of increasing amounts of the XylR protein. The shift of the DNA band for all three target sites was essentially complete at XylR concentration above 0.7 μM. The band shift was suppressed in the presence of 400-fold excess unlabeled DNA fragments but not in the presence of non-specific competitor, salmon sperm DNA (Figure 8). No binding was observed for the negative control DNA segment without XylR-binding DNA motif (see Additional file 4). These observations provided an experimental confirmation of the target XylR-binding sites and XylR-regulated genes tentatively identified by comparative genomic techniques (as described in the previous section).

## Discussion

The comparative genomics analysis and metabolic reconstruction of the xyloside and xylose metabolic subsystem across a broad range of Firmicutes, including *C. acetobutylicum *and related species from Bacilli and Clostridia classes, revealed a remarkable pattern of conservation and variation. The role of this subsystem in utilizing exogenous sources of xylose, and its overall topology (Figure 1) including uptake and degradation of xyloside, xylose transport, and the biochemical conversion of xylose to xylulose 5-P, is preserved in all of the analyzed species. However, nearly all these aspects of its implementation are associated with significant variations such as: (i) existence of alternative transcriptional regulators and regulatory DNA signals; (ii) presence or absence of xyloside utilization machinery and variations therein; (iii) alternative mechanisms of xylose uptake; and (iv) nonorthologous gene displacement for a key enzymatic step of the xylose catabolism, xylose isomerase (Figure 3).

Based on the subsystem reconstruction and the genome context analysis, we have identified a novel xylose isomerase, XylA-II that is not homologous to previously characterized XylA. Moreover, the gene encoding xylulokinase, XylB, was unambiguously identified in all of the analyzed Firmicutes species. These functional predictions were experimentally validated in *C. acetobutylicum *by a combination of genetic and biochemical techniques. The observed low specific activity of XylA-II and XylB from *C. acetobutylicum *is consistent with the relatively slow growth on xylose, suggesting that these two enzymatic reactions may be rate-limiting steps of xylose utilization in this organism. It is interesting that unlike in *C. acetobutylicum *and *C. saccharolyticus *where XylA-II is the only xylose isomerase, both XylA-I and XylA-II are present in *C. beijerinckii *and *C. phytofermentans*. The exact interpretation of the observed functional redundancy in these two organisms requires further investigation.

The genes encoding xylose proton-symporter, XylT, were tentatively identified in several genomes of Firmicutes based on analysis of conserved operons and regulons. This prediction was experimentally assessed by mutagenesis of the inferred gene in *C. acetobutylicum *and by genetic complementation in *E. coli*. The results allow us to conclude that XylT is involved in xylose transport in *C. acetobutylicum*, although there may be additional, unknown mechanism for xylose transport. Despite the differences in cell wall structure between Gram-positive and Gram-negative bacteria, XylT from *C. acetobutylicum *was recognized by the cell sorting machinery of *E. coli *and functioned properly to complement xylose symporter deficiency. It is important to emphasize that although the performed experiments provided strong support for the predicted functional assignment of XylT, additional studies are necessary to establish its substrate specificity and kinetic parameters, as well as to elucidate the mechanism of XylT-independent xylose uptake.

Based on the genome context analysis, the genes encoding the xylose regulator, XylR, were unambiguously assigned in all of the analyzed species. Comparative analysis of upstream regions of the xylose and xyloside utilization genes allowed identification of candidate XylR-binding sites and reconstruction of XylR regulons. A novel XylR-binding DNA motif, which is exceptionally distinct from the DNA motif known for *Bacillus *XylR, was identified in *C. acetobutylicum*, *C. beijerinckii*, and *A. metalliredigens*. Experimental validation was performed by EMSA using purified recombinant XylR protein. The results confirmed the proposed gene assignment of the xylose regulator and the predicted XylR-binding sites in *C. acetobutylicum*. In addition to XylR regulation, expression of *xyl *genes in *Bacillus *is also subject to catabolite repression mediated by the transcriptional factor CcpA that binds to the catabolite responsive element (*cre*) [34]. It has been reported that xylose metabolism in *Clostridium *was inhibited in the presence of glucose [16]. Studies on the involvement of CcpA and *cre *in regulation of *xyl *genes and the contribution of XylR to glucose repression in *Clostridium*, are now underway.

Since pentose sugars (*i.e*. xylose and xyloside) are the most abundant carbohydrate in the hemicellulose of lignocellulosic materials such as hardwoods and crop residues, the efficient utilization of pentose sugars offers the opportunity to significantly reduce the cost of solvent fermentation processes. However, before this study very little was known about the machinery for xylose and xyloside utilization in solventogenic clostridia, although some of the strains were shown previously to have this catabolic potential [17]. A subsystem-based approach applied in this study has allowed us to significantly improve the quality of gene annotations and to accurately infer metabolic and regulatory networks associated with xylose and xyloside utilization in *Clostridium*. Key conjectures about important aspects of this subsystem were validated by focused genetic and biochemical experiments in the model system of *C. acetobutylicum *ATCC 824, although other functional predictions have yet to be experimentally verified. This study paves the way for genetically engineering the xylose pathway in solventogenic clostridia to enhance its capability of xylose utilization.

## Conclusions

We reconstructed the xylose and xyloside utilization pathway and XylR regulons in 24 Firmicutes species by using comparative genomics techniques. It allowed us to discover a novel xylose isomerase that is not homologous to previously characterized xylose isomerase, unambiguously assign the genes encoding the xylulokinase and XylR, and tentatively identify several genes involved in xylose transport and xyloside uptake. The key functional predictions were further experimentally verified in *C. acetobutylicum *through genetic and biochemical techniques. XylR regulons were reconstructed by identification and comparative analysis of XylR-binding sites upstream of xylose utilization genes. We identified and experimentally validated a novel XylR-binding DNA motif in Clostridiales, which is exceptionally distinct from the DNA motif known for *Bacillus *XylR. These findings provided an accurate and comprehensive understanding of xylose metabolism and its regulation in the diverse species of Firmicutes.

## Methods

### Bioinformatics analysis

#### (i) Genome resources and bioinformatics tools

Complete genomes of bacteria from the Firmicutes analyzed in this study were uploaded from GenBank http://www.ncbi.nlm.nih.gov/Genbank/. These and other related genomes used for comparative analysis are integrated in the SEED genomic database http://theseed.uchicago.edu/FIG/index.cgi. Functional coupling of genes via clustering on the chromosome and distribution of genes in the genomes were analyzed using the integrated SEED tools. For analysis of protein families, we used the ClustalX [35] and PHYLIP [36] programs that construct multiple sequence alignments and maximum likelihood phylogenetic trees, respectively. The SignalX program and the Genome Explorer software were used for identification of conserved DNA motifs of transcriptional regulators and genome scanning for candidate DNA-binding sites, respectively [37]. Sequence logos for regulatory motifs were constructed using WebLogo package version 2.6 http://weblogo.berkeley.edu/[38].

#### (ii) Subsystem encoding and genome context analysis

A set of subsystems-based genomic annotations and metabolic reconstruction tools implemented in the SEED were used to capture the existing knowledge of xylose utilization pathways and to tentatively project it to a broader collection of bacteria with completely sequenced genomes. This approach was previously applied for the analysis of various metabolic subsystems and for gene and pathway discovery in a broad range of species [39-41]. Briefly, a subsystem is initiated by defining a list of functional roles (enzymes, transporters, regulators) associated with xylose utilization. This information is obtained by review of pathway-reaction-compound information available in public resources such as KEGG http://www.genome.jp/kegg/ and literature related to xylose metabolism, mostly the studies in *Bacillus *spp. [27,42] and *L. lactis *[23]. In this study we have focused on the xylose transport and conversion of xylose to xylulose 5-P placed in a broader functional context with feeding (xyloside degradation and uptake). Subsystem expansion from model species to other bacteria is accomplished by addition of increasingly distant genomes and orthology-based projection of gene annotations. The phylogenetic boundaries were limited to the Firmicutes phylum that consist mainly of Bacilli and Clostridia classes. The subsystems-based approach to genome analysis and the extensive use of a *genome context *(clustering on the chromosome, phylogenetic profiling, and shared regulatory sites) allowed significant improvement of the accuracy of gene functional assignment and pathway reconstruction. The results of this analysis are captured in the SEED subsystem "Xylose utilization" available at http://theseed.uchicago.edu/FIG/subsys.cgi.

#### (iii) Regulatory signals and regulons

To identify candidate DNA-binding sites of XylR regulators, we started from a set of upstream regions of potentially co-regulated genes in small subgroups of the Bacilli/Clostridia genomes according to the phylogenetic tree of XylR proteins. The training sets included the upstream regions of known XylR targets in *B. subtilis *and/or their orthologs in other XylR-encoding genomes [22,27,43]. An iterative motif detection procedure implemented in the program SignalX was used to identify common regulatory DNA motifs in the training sets and to construct the motif recognition profiles (for a recent review see [44]). The constructed recognition profiles were used to scan a subset of the Bacilli/Clostridia genomes encoding XylR orthologs from the same subgroup on the phylogenetic tree. Positional nucleotide weights in the recognition profile and *Z-*scores of candidate sites were calculated as the sum of the respective positional nucleotide weights. Genome scanning for additional candidate XylR-binding sites was performed using the Genome Explorer software. The threshold for the site search was defined as the lowest score observed in the training set. This analysis produced gene sets with candidate regulatory sites in the upstream regions.

### Bacterial strains, plasmids, and reagents

*E. coli *strains DH5α (Invitrogen, Carlsbad, CA) and ER2275 [45] were used for gene cloning, and BL21(DE3) (Gibco-BRL, Rockville, MD) was used for protein overexpression. *E. coli *K-12 knockout mutants Δ*xylA *(Δb3565), Δ*xylB *(Δb3564) and Δ*xylE *(Δb4031) from the Coli Genetic Stock Center [46] were used for complementation analysis. *C. acetobutylicum *ATCC 824 wild-type and mutant strains were used for analysis of growth phenotype on xylose. The pSY6 vector [47] was used for gene disruption in *C. acetobutylicum*, and pUC118 (Novagen) and pET28a (Novagen) vectors were used for protein expression in *E. coli*. Enzymes for DNA manipulations and PCR were from Fermentas, and plasmid purification kits were from Axygen Biotechnology (Hangzhou, China). Antibiotics, buffer components, and all reagents for enzymatic assays were purchased from Sigma-Aldrich.

### Gene disruption in *C. acetobutylicum *and phenotype analysis

Gene disruption in *C. acetobutylicum *ATCC 824 was performed by using group II intron-based Targetron technology as described previously [47]. Briefly, respective 350 bp fragments for retargeting introns to insert CAC2610 (*xylA-II*), CAC2612 (*xylB*), and CAC1345 (*xylT*) genes were generated by one-step assembly PCR reaction using the primers shown in Additional file 5 according to TargeTron™ gene knockout system (Sigma). The PCR products were then digested and ligated to a targetron plasmid pSY6, yielding the pSY6-*xylA-II*, pSY6-*xylB*, and pSY6-*xylT*. These plasmids were methylated *in vivo *in *E. coli *ER2275 (pAN1) [45] and electroporated into *C. acetobutylicum *ATCC 824, respectively. The transformants were selected on CGM plates with 50 μg ml^-1 ^erythromycin. The resulting mutants with intron insertion of *xyl *genes were confirmed by PCR (see Additional file 3).

For phenotype growth assays *C. acetobutylicum *ATCC 824 wild-type and mutant strains were pre-cultured on CGM medium [48] to late-exponential growth phase, and washed twice using P2 minimal medium [49] without any carbon sources. The cultures were started with the same optical density at 600 nm (OD_600 nm_~0.04), and performed at 37°C in triplicates in 100 ml of P2 minimal medium supplemented with 2, 5, or 20 g l^-1 ^xylose as the sole carbon source. Cell growth was monitored spectrophotometrically at 600 nm. Xylose was quantified by high-pressure liquid chromatography with a model 1200 instrument (Agilent) equipped with a Waters Sugar-Pak I column and a refractive index detector. Double distilled water was used as the mobile phase at a flow rate of 0.6 ml min^-1^, and the column was operated at 70°C.

### Gene cloning in *E. coli *and complementation analysis

The full-length coding regions of CAC2610 (*xylA-II*), CAC2612 (*xylB*), and CAC1345 (*xylT*) from *C. acetobutylicum *ATCC 824 were amplified using the primers shown in Additional file 5. PCR amplification was performed using *C. acetobutylicum *ATCC 824 genomic DNA. PCR fragments were cloned into the pUC118 expression vector digested by *Bam*HI and *Pst*I. The resulting plasmids were transformed into *E. coli *Δ*xylA*, Δ*xylB*, or Δ*xylE *knockout mutants for the complementation analysis. The empty vector was expressed in the same strain and used as a negative control.

The *xylA-II*, *xylB*, and *xylT *genes from *C. acetobutylicum *were expressed constitutively under control of the *lac *promoter in *E. coli *Δ*xylA*, Δ*xylB*, and Δ*xylE*, respectively. Briefly, cells were pre-cultured on Luria-Bertani medium to mid-exponential growth phase, washed twice, and diluted to the same optical density (OD_600 nm_~0.04) using the minimal medium without any carbon sources. Complementation experiments were carried out on modified MacConkey agar plates [50] supplemented with 10 g l^-1 ^xylose or on M9 minimal medium containing 5 g l^-1 ^xylose. After incubation at 37°C for 24 h, the colonies of cells with the ability in xylose utilization showed red color on MacConkey agar plates, whereas the colonies of cells unable to utilize xylose keep yellow.

### Protein overexpression and purification

For protein overexpression, the CAC2610 (*xylA-II*), CAC2612 (*xylB*), and CAC3673 (*xylR*) genes were PCR-amplified (primers shown in Additional file 5) and cloned into the expression vector pET28a. The recombinant proteins were overexpressed as N-terminal fusions with a His_6 _tag in *E. coli *BL21(DE3). The cells were grown on LB medium to an optical density at 600 nm of 0.8 at 37°C, induced by 0.2 mM isopropyl-β-D-thiogalactopyranoside, and harvested after 12 h shaking at 20°C. Protein purification were performed using a rapid nickel-nitrilotriacetic acid (Ni-NTA) agarose minicolumn protocol as described previously [51]. Briefly, harvested cells were resuspended in 20 mM HEPES buffer (pH 7.0) containing 100 mM NaCl, 0.03% Brij-35, and 2 mM β-mercaptoethanol supplemented with 2 mM phenylmethylsulfonyl fluoride and a protease inhibitor cocktail (Sigma-Aldrich). Lysozyme was added to a concentration of 1 mg ml^-1^, and the cells were lysed by freezing-thawing, followed by sonication. After centrifugation at 16,000 g, the Tris-HCl buffer (pH 8.0) was added to the supernatant (final concentration, 50 mM), and it was loaded onto a Ni-NTA agarose column (0.2 ml). After bound proteins were washed with the starting buffer containing 1 M NaCl and 0.3% Brij-35, they were eluted with 0.3 ml of the starting buffer containing 250 mM imidazole. The buffer was then changed to 20 mM HEPES containing 2 mM DTT, 0.5 mM EDTA and 150 mM NaCl by using Bio-Spin columns (Bio-Rad). In all three cases, soluble proteins were obtained with high yield (>1 mg from a 50-ml culture) and purified to >90% by sodium dodecyl sulfate-polyacrylamide gel electrophoresis (see Additional file 6).

### *In vitro *enzymatic assays

#### (i) Xylose isomerase activity

Xylose isomerase activity was assayed by using the colorimetric method as described previously [52]. Briefly, 4-8 μg of purified enzyme was added to 100 μl of 50 mM sodium phosphate buffer (pH 6.8) containing 1 mM MnCl_2_, 5 mM D-xylose, and incubated for 20 min at 37°C. Formation of D-xylulose was monitored by using the cysteine-sulfuric acid-carbazole method and reading the absorbance at 540 nm [52]. The concentration of xylulose was determined from a standard curve which was made using different concentrations of xylulose. No activity was detected in the control experiments in which another gene (CAC2612 or CAC3673) was expressed in the same vector and purified in parallel.

#### (ii) Xylulokinase activity

Xylulokinase activity was assayed by coupling the formation of ADP to the oxidation of NADH to NAD^+ ^via pyruvate kinase and lactate dehydrogenase and monitored at 340 nm. Briefly, 3-6 μg of purified enzyme was added to 500 μl of 50 mM Tris buffer (pH 7.5) containing 10 mM MgSO_4_, 1.2 mM ATP, 1.2 mM phosphoenolpyruvate, 0.3 mM NADH, 1.2 U of pyruvate kinase, 1.2 U of lactate dehydrogenase, and 1 mM D-xylulose. The change in NADH absorbance was monitored at 340 nm by using a Beckman DU-800 spectrophotometer. A NADH extinction coefficient of 6.22 mM^-1 ^cm^-1 ^was used for rate calculation. No activity was detected in the control experiments in which another gene (CAC2610 or CAC3673) was expressed in the same vector and purified in parallel.

### Analysis of XylR-DNA interactions by EMSA

Interaction of purified recombinant XylR protein with its DNA motif was assessed by EMSA. The 180-bp DNA fragments from the upstream region of *xyl *genes were PCR-amplified from *C. acetobutylicum *ATCC 824 genomic DNA using the primers shown in Additional file 5. One of the primers was 5'-biotinylated by Sangon Corp. (Shanghai, China). The PCR products were purified with the PCR purification kit, and their concentration was determined spectrophotometrically.

For EMSA the biotin-labeled DNA (1 nM) was incubated with the indicated amount of purified XylR protein in 20 μl of binding buffer containing 10 mM Tris (pH 7.5), 50 mM KCl, 1 mM DTT, 2.5% glycerol, 5 mM MgCl_2_, and 0.05% NP-40. After 20 min incubation at 28°C, the reaction mixture was electrophoresed at 4°C on a 6% native polyacrylamide gel in 0.5× Tris-borate-EDTA for 2 h at 80 V. The gel was electrophoretically transferred onto a nylon membrane (Millipore, Billerica, MA) and fixed by UV cross-linking. Biotin-labeled DNA was detected with the LightShift Chemiluminescent EMSA kit (Pierce, Rockford, IL). Specificity of the XylR-DNA interactions was tested by including a 400-fold molar excess of non-biotinylated target DNA (specific competitor) and 2 μg salmon sperm DNA (non-specific competitor) in binding reaction mixtures.

## Authors' contributions

CY and WJ conceived and supervised the research, and wrote the manuscript. YG carried out the mutagenesis and genetic complementation experiments. YD performed the EMSA and biochemical analysis. CR and ZS participated in the growth experiments. DAR contributed to the reconstruction of XylR regulons. WZ contributed to the development of the manuscript. SY contributed to the design of the study. All authors read and approved the final manuscript.

## Supplementary Material

Additional file 1 **Occurrence and features of genes involved in xylose and xyloside utilization in Firmicutes**. (A) Distribution of genes involved in xylose and xyloside utilization in Firmicutes. (B) Occurrence of xylose pathway genes in all species of Firmicutes with completely sequenced genomes. Click here for file

Additional file 2 **Candidate DNA-binding sites of xylose regulator XylR in the genomes of the Firmicutes**. Candidate DNA-binding sites of xylose regulator XylR in the genomes of the Firmicutes. Click here for file

Additional file 3 **Confirmation of the respective *C. acetobutylicum *mutants with inactivated *xylA-II*, *xylB*, or *xylT *genes by PCR**. Confirmation of the respective *C. acetobutylicum *mutants with inactivated *xylA-II*, *xylB*, or *xylT *genes by PCR. The genes were disrupted by inserting an intron. Click here for file

Additional file 4 **EMSA to assess the interactions of *C. acetobutylicum *xylose regulator XylR with its cognate DNA signals**. EMSA to assess the interactions of *C. acetobutylicum *xylose regulator XylR with its cognate DNA signals. Each of the three 180-bp target DNA fragments (1 nM) from the upstream region of CAC2611-*xylA-II*, *xylB*, and *xylR *genes, respectively, was incubated for 20 min at 28°C with increasing concentrations of XylR protein (0-0.7 μM). Salmon sperm DNA (2 μg) was added to all binding reaction mixtures as a non-specific competitor. No binding of XylR was observed for the DNA segment from the upstream region of gene CAC1705 that is unrelated to carbon metabolism and used as a negative control. Click here for file

Additional file 5 **Primers used in this study**. Primers used in this study. Click here for file

Additional file 6 **Purified recombinant xylulokinase (XylB), xylose isomerase (XylA-II), and xylose regulator (XylR) from *C. acetobutylicum***. Purified recombinant xylulokinase (XylB), xylose isomerase (XylA-II), and xylose regulator (XylR) from *C. acetobutylicum*. Proteins (1-2 μg each) were purified by Ni-NTA mini-column. Click here for file
