# Supplementary material for: Reconstruction of xylose utilization pathway and regulons in Firmicutes
Source: BMC Genomics. 2010 Apr 21;11:255. doi: 10.1186/1471-2164-11-255 (PMC2873477; doi:10.1186/1471-2164-11-255)
Supplement: Additional file 3 — Confirmation of the respective C. acetobutylicum mutants with inactivated xylA-II, xylB, or xylT genes by PCR. Confirmation of the respective C. acetobutylicum mutants with inactivated xylA-II, xylB, or xylT genes by PCR. The genes were disrupted by inserting an intron. [file 1471-2164-11-255-S3.DOC]

***xylA***

***xylA*+intron**

**Δ*xylA***


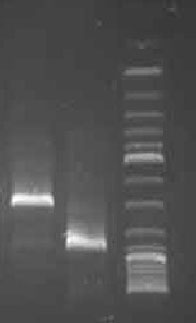


**WT**

**M**

***xylB*+intron**

***xylB***


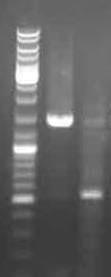


**WT**

**M**

**Δ*xylB***

**Δ*xylT***

**WT**

**M**


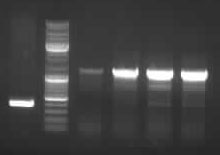


***xylT***

***xylT*+intron**

**Additional file 3.** Confirmation of the respective *C. acetobutylicum* mutants with inactivated *xylA-II*, *xylB*, or *xylT* genes by PCR. The genes were disrupted by inserting an intron.
