# Supplementary material for: Reconstruction of xylose utilization pathway and regulons in Firmicutes
Source: BMC Genomics. 2010 Apr 21;11:255. doi: 10.1186/1471-2164-11-255 (PMC2873477; doi:10.1186/1471-2164-11-255)
Supplement: Additional file 4 — EMSA to assess the interactions of C. acetobutylicum xylose regulator XylR with its cognate DNA signals. EMSA to assess the interactions of C. acetobutylicum xylose regulator XylR with its cognate DNA signals. Each of the three 180-bp target DNA fragments (1 nM) from the upstream region of CAC2611-xylA-II, xylB, and xylR genes, respectively, was incubated for 20 min at 28°C with increasing concentrations of XylR protein (0-0.7 μM). Salmon sperm DNA (2 μg) was added to all binding reaction mixtures as a non-specific competitor. No binding of XylR was observed for the DNA segment from the upstream region of gene CAC1705 that is unrelated to carbon metabolism and used as a negative control. [file 1471-2164-11-255-S4.DOC]

**Additional file 4.** EMSA to assess the interactions of *C. acetobutylicum* xylose regulator XylR with its cognate DNA signals. Each of the three 180-bp target DNA fragments (1 nM) from the upstream region of CAC2611-*xylA-II*, *xylB*, and *xylR* genes, respectively, was incubated for 20 min at 28ºC with increasing concentrations of XylR protein (0-0.7μM). Salmon sperm DNA (2 μg) was added to all binding reaction mixtures as a non-specific competitor. No binding of XylR was observed for the DNA segment from the upstream region of gene CAC1705 that is unrelated to carbon metabolism and used as a negative control.
