# Supplementary material for: Reconstruction of xylose utilization pathway and regulons in Firmicutes
Source: BMC Genomics. 2010 Apr 21;11:255. doi: 10.1186/1471-2164-11-255 (PMC2873477; doi:10.1186/1471-2164-11-255)
Supplement: Additional file 5 — Primers used in this study. Primers used in this study. [file 1471-2164-11-255-S5.DOC]

**Additional file 5.** Primers used in this study

| Gene disruption in *C. acetobutylicum* | | |
| --- | --- | --- |
| Δ*xylA-II* (CAC2610) | IBS: 5′-AAAACTCGAGATAATTATCCTTACTCTTCCCATAAGTGCGCCCAGATAGGGTG-3′  EBS1d: 5′-CAGATTGTACAAATGTGGTGATAACAGATAAGTCCCATAATCTAACTTACCTTTCTTTGT-3′  EBS2: 5′-TGAACGCAAGTTTCTAATTTCGGTTAAGAGTCGATAGAGGAAAGTGTCT-3′ | The primer IBS, EBS1d and EBS2, which were designed using the InGex Intron Prediction Program ([www.Sigma-Aldrich.com/Targetronaccess](http://www.Sigma-Aldrich.com/Targetronaccess)), were used to amplify the targetron target sequence. |
| Δ*xylB* (CAC2612) | IBS: 5′-AAAACTCGAGATAATTATCCTTAATAGGCAATGGAGTGCGCCCAGATAGGGTG -3′  EBS1d: 5′- CAGATTGTACAAATGTGGTGATAACAGATAAGTCAATGGAATTAACTTACCTTTCTTTGT-3′  EBS2: 5′-TGAACGCAAGTTTCTAATTTCGGTTCCTATCCGATAGAGGAAAGTGTCT -3′ |
| Δ*xylT* (CAC1345) | IBS: 5′- AAAACTCGAGATAATTATCCTTAATGGTCGAACTAGTGCGCCCAGATAGGGTG-3′  EBS1d: 5′- CAGATTGTACAAATGTGGTGATAACAGATAAGTCGAACTATTTAACTTACCTTTCTTTGT -3′  EBS2: 5′- TGAACGCAAGTTTCTAATTTCGGTTACCATCCGATAGAGGAAAGTGTCT -3′ |
| Gene cloning in *E. coli* and complementation analysis | | |
| pUC118::CAC2610 | 5′-ggatcctATGAATAATACACCAAAAT-3′  5′-ctgcagTTACTCAAAAGGATTTTCTG-3′ | Introduced restriction sites (BamHI for the 5'-end and Pst I for the 3'-end) are underlined. |
| pUC118::CAC2612 | 5′-ggatcctGTGAGGTATTTATTAGGTAT-3′  5′-ctgcagTTATATATCTTTTATTTTAG-3′ |
| pUC118::CAC1345 | 5′-ggatcct ATGAATAAAAAAATATCTCCAGCAC  5′-ctgcag CTACTCATTTAATCCTCTAACTTTT |
| Protein overexpression and purification | | |
| pET28a::CAC2610 | 5′-ccgggaattccatATGAATAATACACCAAAATTAAAATTAG-3′  5′-caagacgtcgacTTACTCAAAAGGATTTTCTGTTTTA-3′ | Introduced restriction sites (NdeI and BamHI for the 5'-end and SalI for the 3'-end) are underlined. |
| pET28a::CAC2612 | 5′-cgcggatccGTGAGGTATTTATTAGGTATAGACG-3′  5′-caagacgtcgacTTATATATCTTTTATTTTAGGATAAGC-3′ |
| pET28a::CAC3673 | 5′-gggcgcggatccATGTTTGATATAGACCAGAATTCCA-3′  5′-caagacgtcgacTCAAATTACATCTTGTTCAATTAAA-3′ |
| Amplification of 180-bp DNA fragments from the upstream region of genes for EMSA | | |
| CAC2611-2610  (CAC2611-*xylA-II*) | 5'-TTCAGCTTATCCTAAAATAAAAGATATATA-3′  5'-CTCCCCATATAATTTAAATTGGTTT-3′ |  |
| CAC2612 (*xylB*) | 5'-GAAAATTTAAGGGCAGTTTTTTTTA-3'  5'-AATCAAACCCCCTTAATTTTAAATA-3' |  |
| CAC3673 (*xylR*) | 5'-ACGTAAGTGAAGTAAAGATATATAATTG-3′  5'-CACCAACCTTAATCACTAGTATTTT-3' |  |
| CAC1705 (negative control) | 5'-GATTAAggatccGCTGTTAAAAATACTAAAAC-3′  5'-TGATTTTgtcgacTTTCATTTAAAATACCTCC-3' |  |

Nucleotides not present in the original sequence are shown in lowercase
