# Supplementary material for: Reconstruction of xylose utilization pathway and regulons in Firmicutes
Source: BMC Genomics. 2010 Apr 21;11:255. doi: 10.1186/1471-2164-11-255 (PMC2873477; doi:10.1186/1471-2164-11-255)
Supplement: Additional file 6 — Purified recombinant xylulokinase (XylB), xylose isomerase (XylA-II), and xylose regulator (XylR) from C. acetobutylicum. Purified recombinant xylulokinase (XylB), xylose isomerase (XylA-II), and xylose regulator (XylR) from C. acetobutylicum. Proteins (1-2 μg each) were purified by Ni-NTA mini-column. [file 1471-2164-11-255-S6.DOC]

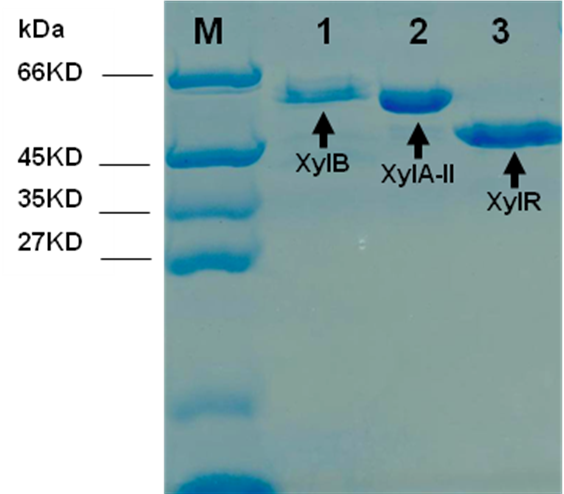


**Additional file 6.** Purified recombinant xylulokinase (XylB), xylose isomerase (XylA-II), and xylose regulator (XylR) from *C. acetobutylicum*. Proteins (1-2 μg each) were purified by Ni-NTA mini-column.
